# Risk perception of coronavirus disease 2019 and career adaptability among college students: the mediating effect of hope and sense of mastery

**DOI:** 10.3389/fpsyg.2023.1210672

**Published:** 2023-08-15

**Authors:** Yadong Ding, Jing Li

**Affiliations:** ^1^Institute of Educational Economics and Management, School of Public Policy and Management, China University of Mining and Technology, Xuzhou, Jiangsu, China; ^2^Department of Emergency Management, School of Public Policy and Management, China University of Mining and Technology, Xuzhou, Jiangsu, China

**Keywords:** risk perception, career adaptability, hope, sense of mastery, COVID-19

## Abstract

The Coronavirus Disease 2019 (COVID-19) pandemic has not only caused widespread economic recession but also had a serious negative impact on the employment of college students. However, little is known about the relationship and mechanisms between the risk perception of COVID-19 and career adaptability. This study aimed to examine whether the risk perception of COVID-19 is associated with career adaptability directly and indirectly through hope and a sense of mastery in college students. A questionnaire survey was conducted among 594 Chinese college students aged 16 to 25, who completed assessments on risk perception of COVID-19, career adaptability, hope, and sense of mastery. The results showed that susceptibility was negatively associated with career confidence, control, and curiosity; moreover, susceptibility indirectly affected career adaptability (including concern, confidence, control, and curiosity) through the sense of mastery; uncontrollable indirectly affected career concern through hope; and uncontrollable indirectly affected career adaptability (including concern, confidence, control, and curiosity) through hope and the sense of mastery. The findings emphasize the role of hope and a sense of mastery in the career adaptability of college students and reveal the necessity of improving hope and a sense of mastery to promote college students' career development. “Implications and limitations are discussed”.

## Introduction

The outbreak of COVID-19 in December 2019 is a career shock for many people around the world (Akkermans et al., 2020). The sudden, persistent, recurrent, and rapidity of transmission of COVID-19 pose a dilemma for the employment of college students (Lan et al., 2020). Due to the long-term impact of the COVID-19 pandemic, the social-economic downturn has reduced the number of jobs, while the number of graduates has increased in recent years, leading to the expansion of employment pressure under the dual influence of “accumulation” and “superposition” (Liu et al., 2022; Cortes and Forsythe, 2023). Studies show that 90% of college students were affected by the suspension of classes, which lead to the reduction of their professional and practical courses in school, and thus their competitiveness in the labor market will be reduced (Sloane and Mavromaras, 2020; Zhuang et al., 2021). The COVID-19 pandemic has a significant effect on the employment environment of college students, bringing new challenges to their career development, and thus leading to more prominent social psychological problems among college students. Multiple pressures such as epidemic prevention, study, and employment, as well as sudden changes in the pace of life, make many students extremely maladjustment, prone to negative emotions, and psychological problems such as tension, anxiety, irritability, loneliness, helplessness, and depression, resulting in a sharp decline in the wellbeing of college students. Although pandemics are difficult to predict and control, research suggests that individuals with better career adaptability can make such career shocks easier to manage and boost wellbeing (Akkermans et al., 2020; Russo et al., 2023; Xu et al., 2023). In the face of increasing uncertainties and changing employment environments, it is particularly important to improve college students' career adaptability so as to enhance their wellbeing.

Career adaptability, as a psychological resource, can help individuals overcome difficulties and setbacks in their career development (Savickas, 1997, 2005), and is a multidimensional structure with four transactional competencies, including concern, control, curiosity, and confidence (Rudolph et al., 2017a,b). No matter how the external environment changes, individuals with a high level of career adaptability can always take the initiative to cope with and maintain a dynamic balance with the environment (Savickas and Porfeli, 2012). Existing studies have confirmed that the career adaptability of college students affects their career development level, and has a close positive causal relationship with their career wellbeing, career decision-making efficacy, perceived employability, and employment status (Maggiori et al., 2013; Guan et al., 2014; Chan and Mai, 2015; Duffy et al., 2015; Atitsogbe et al., 2019). In situations where epidemics are difficult to predict and control, individuals with strong career adaptability have a better quality of employment (He and Yu, 2022). Given the critical role of career adaptability in college students' development, it is of great significance to pay attention to the influencing factors and mechanisms of career adaptability in college students during the COVID-19 pandemic and, in turn, provide insights for facilitating college students' wellbeing and further adaptive development.

A previous study has pointed out the need to explore the influence of the risk perception of COVID-19 on college students' career adaptability (Ding et al., 2020). The career construction theory has indicated that the self-perception of external environmental changes is related to individuals' career development both directly and indirectly through multiple mediators, such as positive psychological capital (Savickas, 1997, 2002, 2005). Numerous researchers have explored factors related to the perception of the external environment as antecedents of career adaptability, such as perceived stress and external support (Zhuang et al., 2021; Parola and Marcionetti, 2022; Fantinelli et al., 2023), but whether or not risk perception of COVID-19 affects college students' career adaptability has garnered less attention. Studying this problem can deepen the understanding of the effect of the risk perception of COVID-19 on the career development of college students.

An individual's career development is caused by a complex and dynamic interaction between environmental and personal factors (Retkowsky et al., 2023). Mariani et al. (2023) pointed out that the accumulation of personal resources can improve a person's employability, increase the ability to respond to the threat of career events, and enhance career adaptability in the case of complex career prospects. Hope and a sense of mastery, as important personal psychological resources, are closely related to college students' career development, wellbeing, and satisfaction (Lachman and Weaver, 1998; Snyder, 2000; Luthans et al., 2010). However, research on the indirect effects of the risk perception of COVID-19 on career adaptability through hope and a sense of mastery has neither been adequately validated nor given sufficient attention. Only a limited number of studies have looked at the impact of stress during the pandemic on career adaptability and the role of positive psychological capital and work volition as potentially important mediators in these relationships (Zhuang et al., 2021). Linking the risk perception of COVID-19, hope and sense of mastery with career adaptability can not only enrich the empirical research but also provide insights for promoting the career development of college students.

### Relationship between the risk perception of COVID-19 and career adaptability

Owing to the COVID-19 pandemic, the economic downturn has reduced job opportunities and increased competition for jobs (Montenovo et al., 2022). However, college students are in a critical period of career development, and the increased uncertainty of the external environment under the influence of the epidemic will inevitably affect their career adaptability. According to the career construction theory, self-perception of external environmental changes can affect individuals' career development and plays an important role in career adaptability (Savickas, 1997, 2002, 2005). The risk perception of COVID-19, as an individual's psychological reflection of the uncertainty of the outbreak of COVID-19, refers to an individual's subjective feelings, experience, and understanding of various objective epidemic risks existing on the outside (Cui et al., 2021), which can be divided into three dimensions (i.e., susceptibility, severity, and uncontrollability). Cui et al. (2021) found that the Chinese public, including college students, showed a higher risk perception during the pandemic. Individuals with a high level of risk perception are more likely to suffer from anxiety, depression, and other problems (Cao et al., 2020; Zhao et al., 2021), thus reducing their career confidence and control. In addition, for the past 3 years, college students in China have mainly taken online courses and were required to complete their graduation online, thus objectively reducing their career development opportunities. Therefore, the risk perception of COVID-19 may be negatively associated with career adaptability.

### Mediating roles of hope and sense of mastery

Hope is regarded as a state of positive motivation, which refers to the expectation of overcoming difficulties reflected by individuals in difficult and unfavorable situations (Stotland, 1969; Snyder, 2000). Hope plays an important role in individual psychological wellbeing (Di Napoli et al., 2019; Shiri et al., 2020). When faced with troublesome situations, a hopeful person will take difficulties as challenges and be able to better deal with difficulties and recover from setbacks (Paul, 2000; Zeng et al., 2022b). The effect of hope on individual wellbeing is not only the buffer effect of negative psychology but also the gain function of positive psychology (Li, 2013). Individuals with a high level of hope are full of expectations for the development and progress of themselves, others, and society, which helps to improve their wellbeing. Hope, therefore, is one of the predictors of career adaptability. Existing studies have found that hopeful individuals tend to perform better in terms of career adaptability and wellbeing, such as proactive career behaviors, life, and job satisfaction (Hirschi, 2014; Buyukgoze-Kavas, 2016; Santilli et al., 2017; Zeng et al., 2022a,b). Similar to hope, a sense of mastery is also a key factor in career adaptation (Blustein et al., 2008; Duffy and Dik, 2009; Duffy, 2010). Sense of mastery refers to a person's sense of efficacy or effectiveness in achieving goals (Lachman and Weaver, 1998). It provides individuals with the motivation to work hard to change their predicament and has adaptive value in the face of adversity (Lachman and Weaver, 1998). Previous evidence suggests that the sense of mastery is associated with positive career commitment, career exploration, and career adaptability (Duffy, 2010; Jia et al., 2020).

Hope and sense of mastery reflect an individual's positive psychological resources, including the confidence to solve challenging tasks, positive recovery in the face of adversity, and a positive outlook on the future (Lachman and Weaver, 1998; Luthans et al., 2010). The conservation of resources theory holds that the depletion of internal resources may cause negative effects (i.e., stress and pessimism), while the preservation of internal resources contributes to the progressive development of the individual (Hobfoll, 2001). When individuals perceive a high level of susceptibility, severity, and uncontrollability of COVID-19, their internal resources may be consumed, thus reducing their hope and sense of mastery. In addition, according to the appraisal theory, when individuals perceive external events as unpredictable, uncertain, and uncontrollable, they tend to feel stressed and have negative emotions (Lazarus, 1991). With its high susceptibility, uncontrollability, and severity, COVID-19 poses a major threat to life. The higher the risk perception level of individuals, the more they believe that the epidemic is uncontrollable, and they are more likely to be infected and may experience more negative emotions (Cao et al., 2020; Zhuang et al., 2021). As a result, college students become pessimistic about their future expectations and lose their sense of mastery over the future, leading to reducing their concern, curiosity, control, and confidence in career development, resulting in poor career adaptability. Taken together, the risk perception of COVID-19 (i.e., susceptibility, severity, and uncontrollability) may be indirectly associated with career adaptability (i.e., concern, control, curiosity, and confidence) through the multiple pathways from hope to sense of mastery.

### The current study

**Figure 1:**
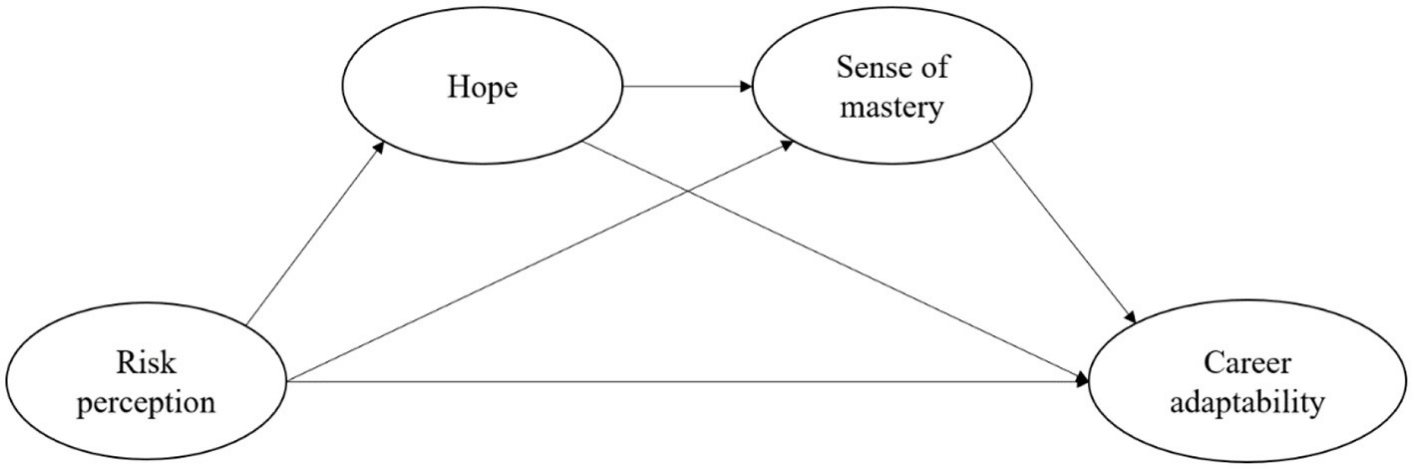
Hypothetical model.

The current study is designed to examine the direct and indirect relationships between the risk perception of COVID-19 (i.e., susceptibility, severity, and uncontrollability) and career adaptability (i.e., concern, confidence, control, and curiosity) among Chinese college students. There are two specific problems in this study. First, does the risk perception of COVID-19 link with career adaptability? We hypothesized that the risk perception of COVID-19 relates to career adaptability. Second, do hope and/or sense of mastery mediate the link from the risk perception of COVID-19 to career adaptability? We hypothesized indirect pathways from the risk perception of COVID-19 to career adaptability through hope and sense of mastery in college students. The abovementioned direct and indirect pathways may be different in different dimensions of risk perception and career adaptability. The conceptual structure model is shown in Figure 1.

## Methods

### Participants and procedure

We conducted a questionnaire survey among undergraduate students at a university in a Chinese city where there was a little outbreak of COVID-19 in October 2022. At that time, students attended the university in person. At that time, students attended the university in person, the campus was a lockdown, and students were asked to stay in their dormitories and attend classes online. Based on convenience sampling, data were gathered in the university through an online survey conducted on the “Questionnaire Star” platform. With the help of the instructors, the questionnaire was distributed through WeChat and QQ groups, and college students were invited to participate in the survey. The formal investigation lasted for 3 days from 25 October to 27 October 2022. A total of 594 questionnaires were collected. The participants ranged in age from 16 to 25 (*M* = 19.23, *SD* = 1.39), of which 197 were male participants and 397 were female participants; they included 190 freshmen, 216 sophomores, 131 junior students, and 57 senior students. At the beginning of the questionnaire, we stated the purpose of this study. Participants were informed that the data were confidential and that they could withdraw from this survey at any time. Informed consent was obtained from all participants in this study.

### Measures

#### Risk perception of COVID-19

The COVID-19 Risk Perception Scale, developed by Cui et al. (2021), was used to measure college students' risk perceptions of COVID-19. A previous study has indicated good reliability and validity of the COVID-19 Risk Perception Scale in Chinese college students (Cui et al., 2021). This scale is a 9-item multidimensional scale measuring three types of risk perception: susceptibility (e.g., “I will be more susceptible than others”), severity (e.g., “Being infected will have very serious health consequences”), and uncontrollability (e.g., “I think the prevalence and spread of this pandemic will be very difficult to control”); each subscale consists of three items. Items were rated on a 5-point scale, ranging from 1 (strongly disagree) to 5 (strongly agree). The mean scores served as their scores. In the present study, the reliability (Cronbach's α) for susceptibility, severity, and uncontrollability subscales was 0.822, 0.838, and 0.750, respectively. Confirmatory factor analysis (CFA) on item levels showed a good fit, χ^2^*/df* = 4.698, *p* < 0.001, RMSEA = 0.079, CFI = 0.985, TLI = 0.959, SRMR = 0.023.

#### Career adaptability

Career adaptability was measured with the Chinese version of the Career Adaptability Scale, which was developed by Soresi et al. (2012). A previous study has indicated good reliability and validity of the career adaptability scale in a Chinese sample (Hou et al., 2012). The Chinese version is a 24-item multidimensional scale with four subscales: concern (e.g., “Realizing that today's choices shape my future”), confidence (e.g., “Performing tasks efficiently”), control (e.g., “Making decisions by myself”), and curiosity (e.g., “Exploring my surroundings”); each subscale consists of six items. The participants responded to all items on a scale from 1 (not strong) to 5 (strongest). The average scores of each subscale served as their scores. In the current study, the reliability for concern, confidence, control, and curiosity was 0.864, 0.914, 0.893, and 0.902, respectively. CFA on item levels showed a good fit, χ^2^*/df* = 3.604, *p* < 0.001, RMSEA = 0.066, CFI = 0.938, TLI = 0.929, SRMR = 0.041.

#### Hope

The State Hope Scale developed by Snyder et al. (1996) was used to measure college students' hope. A previous study indicated good reliability and validity of the Hope Scale among Chinese college students (Li, 2013). The scale has six items (e.g., “At the present time, I am energetically pursuing my goals”). Items were rated on an 8-point scale, ranging from 1 (definitely false) to 8 (definitely true). The mean scores served as the hope scores. In the current study, the reliability was 0.932. CFA on item levels showed a good fit, χ^2^*/df* = 2.417, *p* < 0.001, RMSEA = 0.049, CFI = 0.998, TLI = 0.993, SRMR = 0.009.

#### Sense of mastery

The Sense of Mastery Scale (Lachman and Weaver, 1998) was adapted and translated into Chinese to measure college students' sense of mastery (Li, 2012). A previous study indicated good reliability and validity of the Sense of Mastery Scale in Chinese college students (Li, 2012). The scale has four items (e.g., “When I really want to do something, I usually find a way to succeed at it”). Items were rated on a 7-point scale, ranging from 1 (strongly disagree) to 7 (strongly agree). The mean scores served as the sense of mastery scores. In the current study, the reliability value was 0.86. CFA on item levels showed good fit, χ^2^*/df* = 2.649, *p* < 0.001, RMSEA = 0.053, CFI = 0.997, TLI = 0.991, SRMR = 0.009.

### Statistical analysis

Descriptive statistics and correlation analyses were conducted using SPSS 26.0. Mplus 8.0 was used to conduct structural equation modeling (SEM) to further test the mediating effects. In order to verify the current study's hypotheses, data analysis consisted of two steps. First, the direct effect model was used to examine only the direct paths between risk perceptions of COVID-19 (including susceptibility, severity, and uncontrollability) and career adaptability (including concern, confidence, control, and curiosity). Susceptibility, severity, and uncontrollability were latent variables, all of which were composed of three observation indexes. Career concern, confidence, control, and curiosity were latent variables, all of which were composed of six observation indexes. Second, a chain mediation model was constructed to test the potential mediating roles of hope and sense of mastery. In the SEM analyses, three dimensions of risk perceptions, including susceptibility, severity, and uncontrollability were identified by three observation indexes, respectively. Hope and sense mastery were latent variables composed of six and four observation indexes, respectively. Four dimensions of career adaptability, including concern, confidence, control, and curiosity, were identified by six observation indexes, respectively. It is important to note that individuals' sex, age, and family socioeconomic status may be directly related to their risk perception, hope and sense of mastery, and career adaptability (Li, 2012; Liang et al., 2020; Kiral Ucar et al., 2022). Thus, participants' sex, age, and family socioeconomic status were used as covariates to test the direct and mediating effect models. The fit of the models was assessed using χ^2^*/df* , RMSEA, CFI, TLI, and SRMR. Previous research suggested that the criteria for an acceptable fit are χ^2^*/df* < 5, CFI ≥ 0.90, TLI ≥ 0.90, RMSEA ≤ 0.08, SRMR ≤ 0.08 (Hu and Bentler, 1999). In addition, a non-parametric percentile bootstrap method with high calibration of test force deviation was utilized to estimate the parameters and test the significance of the mediation effect (Wen and Ye, 2014). The number of bootstrap samples was set to 1000, and a 95% confidence interval (CI) was chosen to estimate the significance of the mediation effect. If the 95% CI does not contain zero, a mediation effect was significant.

## Results

### Common method bias, descriptive statistics, and correlation analyses of variables

Since all data in this study were from participants' self-reports, the Harman single factor test was used to conduct common method bias tests before analyzing the results. The results showed that there were six factors with eigenvalues >1, and the variance of the first factor explained was 36.851%, less than the critical criterion of 40% (Zhou and Long, 2004), so there was no significant common method bias.

Table 1 presents all variables' means, standard deviations, and correlations. Correlation analyses showed that susceptibility, severity, and uncontrollability were negatively correlated with hope and sense of mastery; susceptibility was negatively correlated with career confidence, control, and curiosity; severity was negatively correlated with career control; hope, sense of mastery, concern, confidence, control, and curiosity showed positive relationships with each other.

**Table 1:** Mean (M) and standard deviation (SD) of the main variables and correlations between the variables.

	***M* (*SD*)**	**1**	**2**	**3**	**4**	**5**	**6**	**7**	**8**	**9**	**10**
1. Gender	1.67	0.471	—								
2. Age	19.23	1.390	0.113^**^	—							
3. Susceptibility	2.313	0.867	0.078	0.015	—						
4. Severity	2.488	0.966	0.019	−0.001	0.641^***^	—					
5. Uncontrollability	2.663	0.900	0.017	0.024	0.660^***^	0.702^***^	—				
6. Hope	5.416	1.217	−0.053	−0.027	−0.194^***^	−0.190^***^	−0.222^***^	—			
7. Sense of mastery	4.817	0.990	0.001	−0.067	−0.186^***^	−0.109^**^	−0.127^**^	0.684^**^	—		
8. Concern	3.683	0.622	0.053	0.048	−0.076	−0.067	−0.024	0.510^***^	0.543^***^	—	
9. Confidence	3.593	0.630	−0.013	−0.045	−0.115^***^	−0.064	−0.042	0.539^***^	0.591^***^	0.697^***^	—
10. Control	3.846	0.625	−0.020	−0.026	−0.183^***^	−0.124^**^	−0.106^*^	0.432^***^	0.537^***^	0.606^***^	0.725^***^
11. Curiosity	3.758	0.640	−0.009	−0.058	−0.116^**^	−0.079	−0.060	0.456^***^	0.538^***^	0.712^***^	0.704^***^

^*^p <0.05, ^**^p < 0.01, ^***^p < 0.001, male = 1, female = 2.

### The directing effects from risk perceptions of COVID-19 to career adaptability

In order to explore the impact of risk perception on college students' career adaptability, the three sub-dimensions of risk perception (susceptibility, severity, and uncontrollability) were taken as independent variables, and the four dimensions of career adaptability (concern, confidence, control, and curiosity) were taken as dependent variables to establish a direct effect model, with sex, age, and socioeconomic status as covariables. The factor loadings of susceptibility, severity, and uncontrollability were 0.621–0.898, 0.681–0.883, and 0.615–0.794 (*ps* < 0.001), respectively. The factor loadings of concern, confidence, control, and curiosity were 0.576–0.824, 0.730–0.876, 0.557–0.863, and 0.707–0.839 (*ps* < 0.001), respectively. The direct model fit the data well: χ^2^*/df* = 2.676, *p* < 0.001, CFI = 0.933, TLI = 0.923, RMSEA [90%CI] = 0.053 [0.050, 0.056], SRMR = 0.046. The results showed that susceptibility was negatively correlated with career confidence (β = −0.188, *p* = 0.009), control (β = −0.200, *p* = 0.009), and curiosity (β = −0.174, *p* = 0.009); other variables showed non-significant correlation with each other. Sex, age, and socioeconomic status were not significantly correlated with career adaptation (including concern, confidence, control, and curiosity), except that socioeconomic status was positively correlated with career concern, confidence, and curiosity.

### The chain-mediating effects of hope and sense of mastery

**Figure 2:**
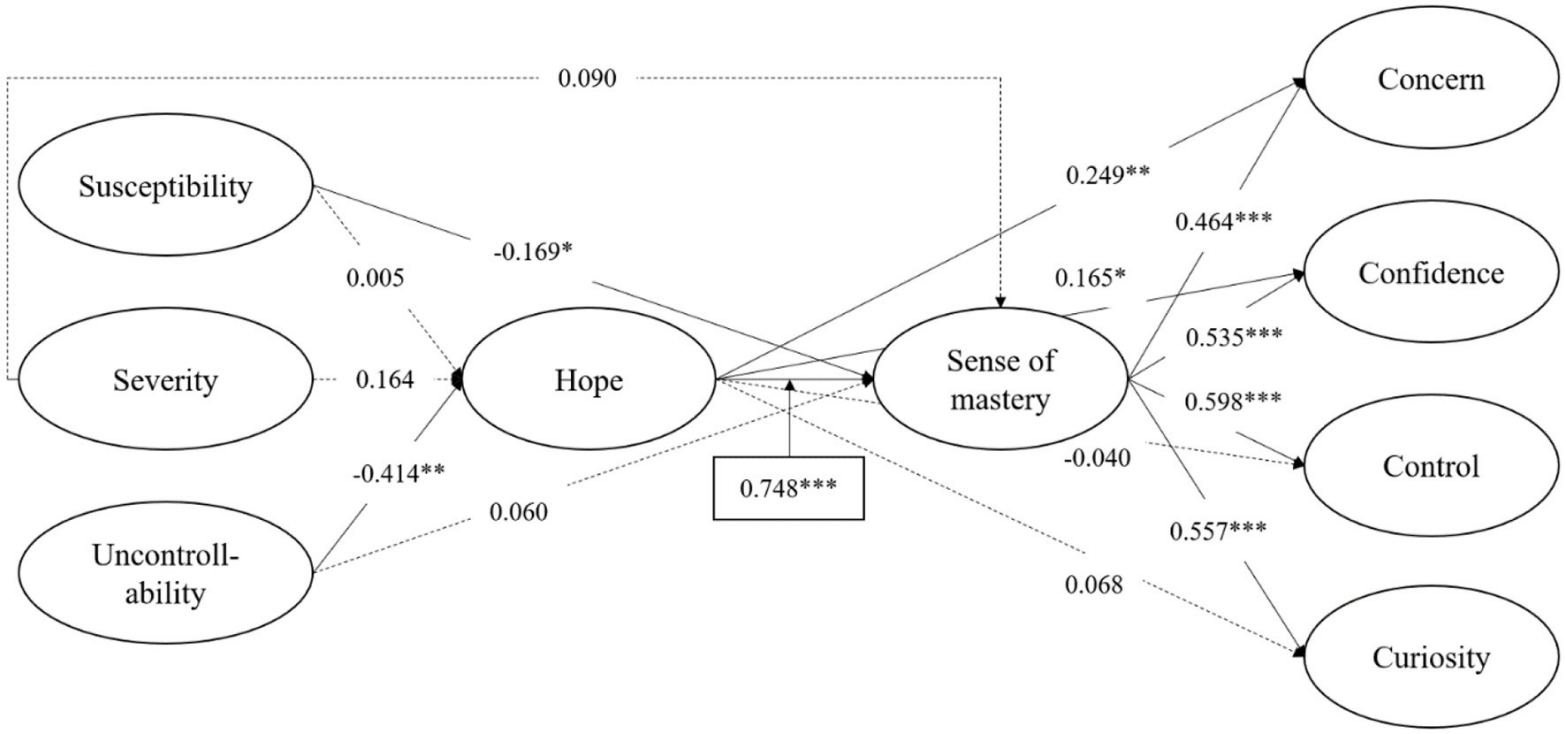
Chain-mediating model. *p < 0.05, ** *p* < 0.01, ***p < 0.001. Standardized coefficients are shown; solid lines represent significant paths, and dotted lines represent non-significant paths.

In order to explore the chain-mediating effects of hope and sense of mastery on the risk perception of COVID-19 and career adaptability, hope and sense of mastery were added to the directing model to establish a chain-mediation model, with sex, age, and socioeconomic status as covariables, as shown in Figure 2. The factor loadings of susceptibility, severity, and uncontrollability were 0.621–0.899, 0.681–0.883, and 0.615–0.792 (*ps* < 0.001), respectively. The factor loadings of concern, confidence, control, and curiosity were 0.577–0.826, 0.731–0.874, 0.556–0.863, and 0.707–0.839 (*ps* < 0.001), respectively. The factor loadings of hope were 0.744–0.932 (*ps* < 0.001). The factor loadings of sense of mastery were 0.709–0.843 (*ps* < 0.001). The chain-mediating model fit the data well: χ^2^*/df* = 2.896, *p* < 0.001, CFI = 0.907, TLI = 0.897, RMSEA [90%CI] = 0.057 [0.054, 0.059], SRMR = 0.054. The results indicated that the sense of mastery played a mediating role in the relationship between susceptibility and career adaptability (including concern, confidence, control, and curiosity); hope played a mediating role in the relationship between uncontrollable and career concern; hope and sense of mastery played a chain-mediating role in the relationship between uncontrollability and career adaptation (including concern, confidence, control, and curiosity). In addition, almost all of the direct pathways were non-significant, except for the direct path from uncontrollable to career concerns (β = 0.378, *p* = 0.042). Sex, age, and socioeconomic status were not significantly correlated with hope, sense of mastery, and career adaptation (including concern, confidence, control, and curiosity), except that socioeconomic status was positively correlated with hope. Bootstrapping results of the mediating effect are shown in Table 2.

**Table 2:** Bootstrapping results of the mediating effect.

**Pathway**	**Est**	**S.E**.	**95%CIs**	**Pathway**	**Est**	**S.E**.	**95%CIs**
SU-HO-CC	0.001	0.023	[−0.039, 0.054]	SU-HO-CO	0.001	0.008	[−0.016, 0.019]
SU-SM-CC	−0.078^*^	0.033	[−0.151, −0.018]	SU-SM-CO	−0.101^*^	0.043	[−0.196, −0.024]
SU-HO-SM-CC	0.002	0.029	[−0.054, 0.061]	SU-OH-SM-CO	0.002	0.038	[−0.070, 0.077]
SE-HO-CC	0.041	0.064	[−0.039, 0.177]	SE-HO-CO	−0.007	0.027	[−0.068, 0.033]
SE-SM-CC	0.042	0.067	[−0.079, 0.183]	SE-SM-CO	0.054	0.083	[−0.118, 0.222]
SE-HO-SM-CC	0.057	0.072	[−0.055, 0.220]	SE-HO-SM-CO	0.073	0.093	[−0.074, 0.280]
UN-HO-CC	−0.103^*^	0.083	[−0.297, −0.005]	UN-HO-CO	0.017	0.045	[−0.061, 0.115]
UN-SM-CC	0.028	0.078	[−0.127, 0.174]	UN-SM-CO	0.036	0.101	[−0.163, 0.248]
UN-HO-SM-CC	−0.144^*^	0.084	[−0.350, −0.017]	UN-HO-SM-CO	−0.185^**^	0.108	[−0.433, −0.023]
SU-HO-CF	0.001	0.016	[−0.026, 0.036]	SU-HO-CU	0.001	0.009	[−0.017, 0.022]
SU-SM-CF	−0.090^*^	0.038	[−0.175, −0.020]	SU-SM-CU	−0.094^*^	0.041	[−0.182, −0.021]
SU-HO-SM-CF	0.002	0.034	[−0.061, 0.068]	SU-HO-SM-CU	0.002	0.035	[−0.063 0.071]
SE-HO-CF	0.027	0.044	[−0.025, 0.131]	SE-HO-CU	0.011	0.029	[−0.033, 0.080]
SE-SM-CF	0.048	0.075	[−0.093, 0.211]	SE-SM-CU	0.050	0.079	[−0.110, 0.217]
SE-HO-SM-CF	0.066	0.083	[−0.068, 0.256]	SE-HO-SM-CU	0.068	0.089	[−0.068, 0.270]
UN-HO-CF	−0.069	0.062	[−0.224, 0.005]	UN-HO-CU	−0.028	0.049	[−0.132, 0.056]
UN-SM-CF	0.032	0.089	[−0.157, 0.211]	UN-SM-CU	0.033	0.352	[−0.157, 0.232]
UN-HO-SM-CF	−0.166^**^	0.095	[−0.394, −0.022]	UN-HO-SM-CU	−0.173^**^	0.103	[−0.423, −0.021]

SU, susceptibility; SE, severity; UN, uncontrollability; HO, Hope; SM, sense of mastery; CC, concern; CF, confidence; CO, control; CU, curiosity. ^*^p < 0.05, ^**^p < 0.01, and ^***^p < 0.001.

## Discussion

The COVID-19 pandemic has not only caused widespread economic recession but has also had a serious negative impact on the employment of college graduates in China. It is necessary to study the career adaptability of college students during the COVID-19 pandemic, understand the influencing factors and mechanisms of career adaptability, and further provide a scientific basis for the intervention measures of career adaptability. The current study examined whether risk perceptions of COVID-19 were associated with career adaptability directly and indirectly through hope and sense of mastery in college students. The results showed that there were direct pathways from susceptibility to career confidence, control, and curiosity. In addition, indirect pathways were different in different dimensions of risk perception and career adaptability. Specifically, susceptibility indirectly affected career adaptability (including concern, confidence, control, and curiosity) through sense of mastery; uncontrollable indirectly affected career concern through hope; uncontrollability indirectly affected career adaptability (including concern, confidence, control, and curiosity) through hope and sense of mastery.

### The direct influence of the risk perception of COVID-19 on career adaptability

The current study found that susceptibility was negatively associated with career confidence, control, and curiosity in college students. The social cognitive model holds that an individual's risk perception is related to behavioral intention (Lee and Lemyre, 2009). Depending on this model, when people face a public health emergency such as COVID-19, there may be negative emotional and behavioral responses such as anxiety, panic, as well as avoidance behavior (Hai-Dong et al., 2022). The recurrence and uncertainty of the COVID-19 pandemic have an effect on social and economic development and the employment environment. Many companies stop hiring in order to reduce operating costs, resulting in fewer and fewer job opportunities for college graduates, thus reducing college students' confidence in their future career development (Lan et al., 2020). Moreover, when college students have a high level of susceptibility, they will have more inner panic and anxiety, which is not conducive to the individual's active exploration of the surrounding environment and their career role, and curb the exploration of the employment environment and the vision of planning career role, thus reducing their career curiosity. In order to avoid the risk of infection, the reduction of job fairs objectively reduces the employment opportunities for college students, which leads to a feeling of more powerlessness about their future careers, and thus a lower sense of control over their careers. In this way, susceptibility to COVID-19 reduces college students' career confidence, control, and curiosity. The current study shows that the COVID-19 pandemic as a career shock is negative. The occurrence of career shock can be positive or negative, and its impact on one's career can be negative or positive (Hobfoll et al., 2018; Ali and Mehreen, 2022). Akkermans et al. (2020) argued that while the COVID-19 pandemic is a negative shock for most people, in future, it could contribute to more positive outcomes. Future research should consider longitudinal tracking to explore the negative or positive long-term effects of COVID-19 on an individual's career development.

### Risk perception of COVID-19 indirectly affects career adaptability through hope and sense of mastery

The result of the current study suggested that uncontrollable indirectly affected career concerns through hope. COVID-19, as an external risk, is highly uncontrollable in terms of its development trend, virus avoidance, and safety protection (Ding et al., 2020). When college students' perception of the uncontrollable COVID-19 epidemic is enhanced, they will have the perception of being infected with the virus and being enveloped by the virus, which will affect their hope for their desired goals. Psychological stress theory proposes that an individual will produce a series of emotional, behavioral, and physiological stress responses based on personal cognitive assessment in major crisis moments (Lazarus and Folkman, 1984). Because the risk of COVID-19 is uncertain, incomplete information about the severity of the risk of the outbreak can cause psychological and emotional stress and may lead to negative psychological states (Etkin, 2009). Meanwhile, negative information about infectious diseases can lead to irrational fear and stress, and this negative emotional state can trigger pessimistic motivation (Wu, 2022), which in turn weakens the sense of hope. Therefore, when the perceived uncontrollability increases, people will experience a negative mental state, such as anxiety and pessimism, and reduce hope. Hope can be seen as a powerful motivator that drives inner energy to deal with difficulties and challenges (Biassoni et al., 2022). Individuals with a high level of hope tend to show concern for the future and look to the future, and to prepare for future tasks that may arise. Thus, the greater the individual's hope, the higher the career concern. Previous studies have shown that hope contributes to an individual's career concerns (Stevenson et al., 2021; Zeng et al., 2022a,b). Thus, uncontrollability can be indirectly associated with career concerns through hope.

In the context of the COVID-19 pandemic, the competition among college students is becoming more and more fierce, and the pressure in academic, economic, and employment aspects is greater, resulting in an increase in negative emotions, dissatisfaction with reality, and a loss of hope and confidence among college students in future. When individuals do not have a healthy sense of hope, it will hinder individual's goal-setting, resulting in a lack of self-confidence, lack of motivation, and difficulty coping with stress and setbacks. Hope can not only buffer negative emotions but also enhance individual wellbeing. Researchers also point out that even if people are living in very difficult times, as long as there is hope, individuals can experience wellbeing (Li, 2013). This study focuses on the adaptive development of college students from the positive perspective of hope, which not only helps to promote the career adaptability of college students but also has important significance for improving their mental health and wellbeing.

The current study further found that hope and sense of mastery played a chain-mediated role in the relationship between uncontrollability and career adaptation (including concern, confidence, control, and curiosity). Previous studies reported a close relationship between hopelessness and perceived control (Kiral Ucar et al., 2022), which also implies a possible close relationship between hope and sense of mastery. Meanwhile, studies have shown that high school and college students with high levels of sense of mastery have higher levels of career self-efficacy, higher career ambition, greater career determination, and adaptability (Lease, 2004; Millar and Shevlin, 2007; Duffy, 2010). Thus, sense of mastery contributes to career adaptability, which was supported by our findings. Confidence in one's abilities appears to play a key role in maintaining an individual's employability, helping to improve the overall chances of finding a job (Capone et al., 2021). Self-efficacy is a personal resource related to student's wellbeing during the COVID-19 pandemic, which helps to improve students' career adaptability and wellbeing (Zeng et al., 2022a). Moreover, the current study showed that susceptibility was indirectly associated with career adaptability (including concern, confidence, control, and curiosity) through sense of mastery. Previous research has shown that risk perception was negatively associated with sense of mastery (Nordgren et al., 2007; Ruthig et al., 2007; Huang et al., 2023). When individuals perceive risks and threats in the environment, especially those beyond their control, it may lead to a decrease in their sense of mastery (Huang et al., 2023). The findings from the current study further support the important role of hope and sense of mastery in determining career adaptability among Chinese college students.

### Implications

The current study provides some contributions. First, the current findings provide empirical evidence for a theoretical model that suggests there are multiple pathways from perceived risk to career adaptability (i.e., through hope and sense of mastery). This study confirms the role of mediating variables (hope and sense of mastery) as buffers against the negative impact of risk perception on the career adaptability of college students. The current study underscores the importance of hope and a sense of mastery in the relationship between risk perception and career adaptability. Even though college students have a high-risk perception, educators can improve their career adaptability by interfering with their hope and sense of mastery to reduce the negative influence of risk perception on their career adaptability. In the actual mental health education and counseling work, college student management workers should take the initiative to learn more about students' daily life and learning conditions, guide students to adjust their attention bias to external information, pay more attention to positive information, and give students enough care and support to enhance their sense of hope and control for the future, thus effectively reducing the risk of anxiety and depression and promoting the development of student's careers. Second, college is a crucial period for career development, and the current study indicates that hope may be a protective factor and a positive motivator for college students' career adaptability. As an important personality force, hope's core function is to relieve individual negative emotions and improve individual mental health (Snyder, 2000). In the process of guiding students to pursue goals, educators should provide students with specific methods and plans to achieve goals, stimulate students to pursue future goals with stronger motivation, enhance their sense of control, and further promote their pursuit of career goals. Third, the current study indicates that the sense of mastery is a kind of internal resource that can improve the adaptability of a person when she or he is in a disadvantageous situation. In daily education and teaching, educators should guide students to improve their sense of internal mastery, so as to reduce the impact of negative experiences on their development, especially large emergencies. Finally, during the pandemic, due to the uncertainty and uncontrollability of the epidemic, college students showed a high degree of stress perception, including academic, economic, and employment pressure (Zhuang et al., 2021). More psychological problems may occur under the influence of multiple pressures. Therefore, college educators should pay attention to students' perceived stress during the COVID-19 pandemic. To reduce their perception of stress and improve their career adaptability and wellbeing, university administrators should offer students online career planning courses and mental health courses. University administrators can also use positive psychological resources—hope as a tool to improve college students' career adaptability and wellbeing. Flexible intervention programs can also be developed through social media platforms such as WeChat.

### Limitations and future research

There are some limitations to the current study. First, the current study was cross-sectional and did not take into account the changing landscape during the COVID-19 pandemic. People may perceive risks differently in the early and late stages of the outbreak. Future studies should consider tracking the entire process of an emergency and collecting data at multiple time points to further understand how risk perception affects college students' career adaptability, and verify the causal relationships among risk perception, hope, sense of mastery, and career adaptability in college students. Second, the data were collected in China. While the current findings provide useful insight into the role of risk perception on career adaptability during the COVID-19 pandemic, future researchers should be cautious in generalizing the findings to other situations and different countries with different restrictions. Cultural differences may influence subjective assessments of risk. Future studies should further expand the samples from different countries to further verify and extend the conclusions of this study. Third, the 12-item version of the career adaptability scale-short form has gradually been used by different groups in different countries and regions (Song et al., 2023). A very recent study has shown that the career adaptability scale-short form has good reliability and validity in the Chinese sample (Song et al., 2023). Future research should use the career adaptability scale-short form to further explore the influencing factors of career adaptability and improve the career development of college students.

## Data availability statement

The raw data supporting the conclusions of this article will be made available by the authors, without undue reservation.

## Ethics statement

Ethical review and approval was not required for the study on human participants in accordance with the local legislation and institutional requirements. Written informed consent from the patients/participants or patients/participants' legal guardian/next of kin was not required to participate in this study in accordance with the national legislation and the institutional requirements.

## Author contributions

YD was responsible for literature search and article writing. JL was responsible for data collection and analysis. All authors contributed to the article and approved the submitted version.
